# Assessment of chemotherapeutic effects on cancer cells using adhesion noise spectroscopy

**DOI:** 10.3389/fbioe.2024.1385730

**Published:** 2024-05-13

**Authors:** Maximilian Ell, Mai Thu Bui, Seyda Kigili, Günther Zeck, Sonia Prado-López

**Affiliations:** ^1^ Institute of Biomedical Electronics, Faculty of Electrical Engineering and Information Technology, TU Wien, Vienna, Austria; ^2^ Institute of Solid State Electronics, Faculty of Electrical Engineering and Information Technology, TU Wien, Vienna, Austria

**Keywords:** CMOS microelectrode array, cell adhesion noise spectroscopy, colorectal cancer cells, human dermal fibroblasts, anti-cancer therapeutics

## Abstract

With cancer as one of the leading causes of death worldwide, there is a need for the development of accurate, cost-effective, easy-to-use, and fast drug-testing assays. While the NCI 60 cell-line screening as the gold standard is based on a colorimetric assay, monitoring cells electrically constitutes a label-free and non-invasive tool to assess the cytotoxic effects of a chemotherapeutic treatment on cancer cells. For decades, impedance-based cellular assays extensively investigated various cell characteristics affected by drug treatment but lack spatiotemporal resolution. With progress in microelectrode fabrication, high-density Complementary Metal Oxide Semiconductor (CMOS)-based microelectrode arrays (MEAs) with subcellular resolution and time-continuous recording capability emerged as a potent alternative. In this article, we present a new cell adhesion noise (CAN)-based electrical imaging technique to expand CMOS MEA cell-biology applications: CAN spectroscopy enables drug screening quantification with single-cell spatial resolution. The chemotherapeutic agent 5-Fluorouracil exerts a cytotoxic effect on colorectal cancer (CRC) cells hampering cell proliferation and lowering cell viability. For proof-of-concept, we found sufficient accuracy and reproducibility for CAN spectroscopy compared to a commercially available standard colorimetric biological assay. This label-free, non-invasive, and fast electrical imaging technique complements standardized cancer screening methods with significant advances over established impedance-based approaches.

## 1 Introduction

Cancer is a major burden worldwide that affected 19.3 million people in 2021. According to the Global Cancer Observatory (GLOBOCAN), the number of new cancer patients per year is expected to reach 28.4 million in 2040. Among all types of cancer, colorectal cancer (CRC) represents 10% of new cases and is the second cause of cancer death worldwide (Sung et al., 2021). This puts the focus on the development of new therapies for the disease to assess the drug efficacy in a quick and cost-effective way by implementing new technologies.

Bringing a new drug on the market requires a preclinical phase and a clinical trial with high costs. The whole process before approval demands 10–15 years and costs millions of euros (Marchetti and Schellens, 2007). In the preclinical phase, the drug candidates are tested first *in vitro* and then *in vivo*. For five decades, the NCI 60 cell-line screening has been the gold standard for anti-cancer drug development (Chabner, 2016). This panel includes 60 cell lines for nine different types of cancer (leukemia, melanoma, non-small cell lung carcinoma, ovary, brain, breast, kidney, colon, and prostate). The evolution of the drug screening tests based on the NCI 60 cell lines is reviewed in detail elsewhere (Chabner, 2016). In brief, the screening process starts with an initial test of just three of the cell lines. If the tested compounds present growth-inhibiting activity, they will be screened against the full cell panel. The testing approach uses a colorimetric assay (sulforhodamine blue cytotoxicity assay), incubated for 48 h with five different concentrations of the tested drugs. Next, the GI50 (Growth Inhibition of 50%), the TGI (Total Growth Inhibition), and the LC50 (drug concentration with 50% reduction of the measured protein) are calculated (Developmental Therapeutics Program, 2021). Afterwards, an algorithm forms clusters of drugs with the same type of cytotoxic activity. Moreover, the relative expression of hundreds of molecular targets that are known to be present in the cell lines is correlated with the drug sensitivity profile against every drug (Takimoto, 2003). Next, the selected drug candidates are assessed for pharmacokinetic and toxicokinetic behavior *in vivo* and—if successful—they will enter the Phase I clinical trial in humans. Although many compounds enter the clinical trials phase, many are proven not to be efficacious in human subjects later. The reasons for the low ratio of success in the clinic can be multifactorial. On the one hand, the 2D *in vitro* culture system cannot fully represent the tumoral complexity, and on the other hand, the *in vivo* models are biologically different from humans. 3D tumoral models generated with patient-derived material for drug testing are proposed as a more physiological approach for basic cancer research and drug testing and are gaining momentum in many fields, including cancer research. But adapting the drug screening assays from the 2D to the 3D cultures will take some time. However, the 3D models also present drawbacks and are far from perfect. For example, the 3D tumoral models tend to become necrotic in the center after several days in culture due to the lack of a circulatory system (Pugh and Ratcliffe, 2003), which can yield misleading results for drug testing. In this work, we propose the use of CMOS-based microelectrode arrays (CMOS MEAs) in combination with cancer cells growing in 2D on different extracellular matrix (ECM) proteins to assess chemotherapeutic effects on CRC cells using adhesion noise spectroscopy to complement drug screening techniques and reveal features not accomplished by optical readouts.

Monitoring cells electrically and electrochemically offers a label-free (Magar et al., 2021), easy-to-use (Turner, 2013; Mehrotra, 2016), and portable (D. Zhang and Liu, 2016) technique to gain further insight into cell properties that are difficult to obtain by optical density proliferation and cytotoxicity assays [e.g., cell adhesion (Srinivasan et al., 2015)], but suffer from spatial resolution (Wegener et al., 2000). Hence, CMOS-based microelectrode arrays (CMOS MEAs) enable recordings of the cells’ electrical activity with high spatial (few to few tens of µm) and high temporal (up to 20 kHz bandwidth) resolution using thousands of densely packed sensor sites (Müller et al., 2015; Thewes et al., 2016). This new CMOS MEA application allows for the non-invasive probing of the electrical properties of the adhesion cleft of adherent cells either via impedance spectroscopy (Viswam et al., 2018) or cell adhesion noise (CAN) spectroscopy (Zeitler et al., 2011). In impedance spectroscopy, an external (small) AC current is applied and the impedance of the cleft between the cell membrane and the oxide surface and of potentially capacitive cellular components is recorded by the electrode underneath the cell. In CAN spectroscopy, sensing relies on the thermal noise generated by the cleft resistance between the adherent cellular membrane and the capacitive recording site. Adherent non-electrogenic cells are detected by an increase in extracellular voltage fluctuations caused by the cleft resistance between the cell membrane and a field-effect transistor (Voelker and Fromherz, 2006; Zeitler et al., 2011; Zeitler and Fromherz, 2013). Moreover, CAN spectroscopy evaluates the voltage noise of cells adhered to a CMOS MEA in terms of spectral power density (S_V_) without the need for any external stimulation (Eickenscheidt et al., 2012; Bertotti et al., 2014; Stutzki et al., 2016) or perturbation of cells [e.g., contacting with a patch pipette (Im et al., 2018)]. In conclusion, monitoring cell cultures using CAN spectroscopy offers not only label-free but also a non-invasive electrical imaging method with high spatiotemporal resolution for drug screening applications.

To use CAN spectroscopy in a biological setting, we focus on detecting early changes in cell adhesion of the (CRC) cell line HT-29 and non-cancerous human dermal fibroblasts (HDFs) growing in 2D cultures on top of the dielectric oxide of the MEA coated with ECM proteins after treating them with the chemotherapeutic agent 5-Fluoruracil (5-FU) (N. Zhang et al., 2008). Since cancer cells are immortal cells that proliferate abnormally, we correlate adhesive properties and cells’ health status (Tubiana, 1989; Hanahan and Weinberg, 2011; Feitelson et al., 2015). Therefore, cell proliferation is determined by the MEA’s area covered with adherent cells, assuming a loss in cell adhesion due to the cytotoxic anti-cancer drug 5-FU (Akalovich et al., 2021; Fohlen et al., 2021). Our MEA comprises both subcellular resolution (Hierlemann et al., 2011; Viswam et al., 2018; Ell, Zeitler, et al., 2023) and cellular network studies (Amin et al., 2017; Viswam et al., 2017; Angotzi et al., 2018; Abbott et al., 2022; Cojocaru et al., 2022; Emery et al., 2022; 2023). To relate electrical imaging to optical imaging, CAN-based images were overlaid with brightfield microscopy images. Just to verify the accuracy of our technology localizing the cells physically. Moreover, we assessed the cell viability on the MEA with the commercial device CASY Cell Counter and Analyzer (Weinreich et al., 2014; Grintzalis et al., 2017). Besides, both cell types were grown in 96-well plates and quantified using a commercially available colorimetric cell viability assay (Cell Counting Kit-8, CCK-8). The technology presented in this work provides a cell-based, label-free, non-invasive, and fast assay to assess drug development applications in a time-resolved manner.

## 2 Methods

### 2.1 Cell culture 2D models

The colorectal cancer (CRC) cell line HT-29 (ATCC, RRID: CVCL_0320) and the human dermal fibroblast cell line HDF (PELOBiotech GmbH, RRID: CVCL_DP66) were cultivated at 37°C in a 5% CO_2_ atmosphere. The used culture medium consisted of Dulbecco’s Modified Eagle’s Medium (DMEM (1X), Thermo Fisher Scientific Inc.), 10% v/v Fetal Bovine Serum (FBS, heat-inactivated, Thermo Fisher Scientific Inc.), 1% v/v Gibco™ Penicillin Streptomycin (Pen Strep, Thermo Fisher Scientific Inc.) and 1% v/v Gibco™ L-glutamine 200 mM (Thermo Fisher Scientific Inc.). We changed the cell culture medium every 2 days and passaged the cells at 80% confluency.

### 2.2 Coating-dependent cell attachment

Cell-cell and cell-extracellular matrix (ECM) protein interactions are essential for proper tissue development, maintenance, and regeneration. Thus, *in vitro* cells adhere, grow, and perform more physiologically when adhering to surfaces coated with ECM proteins or specific adhesion molecules (Cruz Walma and Yamada, 2020) Both Poly-L-lysine, which is commonly used in neuroscientific applications (Eickenscheidt and Zeck, 2014; Stutzki et al., 2016; Cojocaru et al., 2022) and Collagen type I as the most abundant ECM protein in our body (Murata et al., 1986; Katsuda et al., 1992; Hohenester and Engel, 2002) ensure tight tissue adhesion (Vitale et al., 2018). We therefore addressed the biocompatibility of the cited ECM biomolecules (Prockop and Kivirikko, 1995; Vitale et al., 2018) by analyzing the viability of the cell line HT-29 cultivated in 96-well plates and on CMOS MEAs coated with Poly-L-lysine or Collagen type I.

#### 2.2.1 96-well plate

The Nunc™ MicroWell™ 96-well, Nunclon Delta-Treated, Flat-Bottom Microplates (Thermo Fisher Scientific Inc.) were coated with either 0.01 μg/μL Poly-L-lysine (PLL) (MW 150–300 kDa, Sigma-Aldrich GmbH) or 10% (v/v) Collagen type I (from calfskin, Sigma-Aldrich GmbH) for 2 hours at room temperature. Afterwards, they were washed with 2 × 100 µL PBS (1X) and 1 × 100 μL cell culture medium to remove the excessive coating solution. After coating, we seeded 10,000 cells per well, let the cells sediment and adhere sufficiently overnight, and started to conduct the CCK-8 assay. The medium was exchanged every 2 days to avoid nutrient depletion, building-up of metabolic products, and a lowered pH (Masters and Stacey, 2007).

The Cell Counting Kit-8 (CCK-8) assay (MedChemExpress) was used at 0 h and 48 h after the cells’ seeding, following the provider’s recommendations. Briefly, cells were incubated with 10% CCK-8 for 2 hours, and the optical density (OD) was read at 450 nm with a microplate reader (Byonoy GmbH). To calculate cell viability from OD, we used the formula Cell Viability (%) = (Sample Signal–Blank Signal)/(Control Signal–Blank Signal) × 100. We conducted the experiments independently at least three times, each with 3-5 technical replicates. Statistics were performed using the mean and standard deviation of the OD or Cell Viability with GraphPad Prism 10 (GraphPad Software, RRID: SCR_002798). Unpaired t-tests/ANOVA compared the significant differences between the two experimental groups. The significance level is denoted as ns, not significant, *, *p* ≤ 0.05, **, *p* ≤ 0.01, ***, *p* ≤ 0.001, ****, *p* ≤ 0.0001.

#### 2.2.2 CMOS MEA

We used the CMOS MEA system CAN-Q Station with the biosensing platform CAN-Q Chip (obtained from formerly Venneos GmbH) to record the MEAs (Vallicelli, De Matteis, et al., 2018). To prepare the CMOS MEA for cell seeding, we cleaned it with Tickopur R60 (5% v/v at 80°C, Dr. H. Stamm GmbH Chemische Fabrik), sterilized with 70% v/v ethanol and UV light for 30 min, and rinsed with distilled water and cell culture medium. Next, the recording sites were coated with 50 µL of Collagen or PLL solution of the same concentrations as the 96-well plates. After 2 hours of incubation at room temperature, we rinsed the CMOS MEA with distilled water to withdraw the excessive coating solution. The HT-29 cells were then seeded at a concentration of 20,000 cells/chip in a 50 μL cell-culture-medium suspension by pipetting on the sensor window array. One hour of incubating at 37°C in a 5% CO_2_ atmosphere ensured adequate cell sedimentation on the CMOS MEA’s sensor sites, and next, the CMOS MEA’s culture chambers were filled up with cell culture medium. After letting the cells attach to the sensors, recordings were performed at 0 h, 24 h, 48 h, and 72 h in a bio-safety cabinet Class II at room temperature.

We analyzed the recordings with a custom Python script (Python version 3.9.5, RRID: SCR_008394). An electrical image was generated by the spectral power density S_V_ with image processing techniques using the OpenCV Library (RRID: SCR_015526) (Bradski, 2000). The cell viability status was estimated from the proliferation status: we assume that healthy and proliferative cells cover a larger sensor area following (Witzel et al., 2015). The area covered by cells was calculated using the formula area (%) = Number of Cell-covered Sensors/Total Number of Sensors 100. We performed unpaired t-tests/ANOVA for the statistics of the mean and standard deviation of the area using GraphPad Prism 10 with ns, not significant, *, *p* ≤ 0.05, **, *p* ≤ 0.01, ***, *p* ≤ 0.001, ****, *p* ≤ 0.0001.

### 2.3 Drug testing efficacy

We evaluated the effect of 5-Fluorouracil (5-FU, Sigma-Aldrich GmbH) on HT-29 and HDF. For that, the cell lines were cultivated in 96-well plates and on CMOS MEAs coated with Collagen type I. Recent studies indicate that 5-FU exerts a cytotoxic effect on HT-29 cells (Fohlen et al., 2021), leading to cell apoptosis (Vallicelli, Fary, et al., 2018). We therefore assumed that apoptotic cells (i) generate a lower OD during the CCK-8 assay with lower Cell Viability and (ii) detach from the ECM coating, causing fewer sensors where cells are adhered to, which results in a smaller area of the CMOS MEA sensor array covered by cells.

For these experiments, we coated the CMOS MEAs with 10% (v/v) Collagen type I and seeded both cell lines in separate experiments. HT-29 was seeded at a concentration of 20,000 cells/chip as described before (2.2) and HDF was seeded at a concentration of 2,000 cells/chip. Both cell lines were seeded on the uncoated 96-well plates at a concentration of 10,000 cells/well. After overnight incubation for cell adhesion, we replaced the cell culture medium with a medium containing 0.25 mg/mL of 5-FU. The cells were kept in the 5-FU-supplemented medium for the remaining experimental time (72 h). For the untreated control group, we exchanged the medium at the same time when the drug treatment started. The 5-FU-containing medium was prepared freshly before each treatment with a 1 mg/mL stock solution.

We performed the CCK-8 assay and the CMOS MEA recordings at 0 h, 24 h, 48 h, and 72 h after overnight adhesion. For the statistical analysis (*t*-test and ANOVA), we used the GraphPad Prism 10 software with ns, not significant, *, *p* ≤ 0.05, **, *p* ≤ 0.01, ***, *p* ≤ 0.001, ****, *p* ≤ 0.0001.

### 2.4 CMOS MEA recording

**FIGURE 1:**
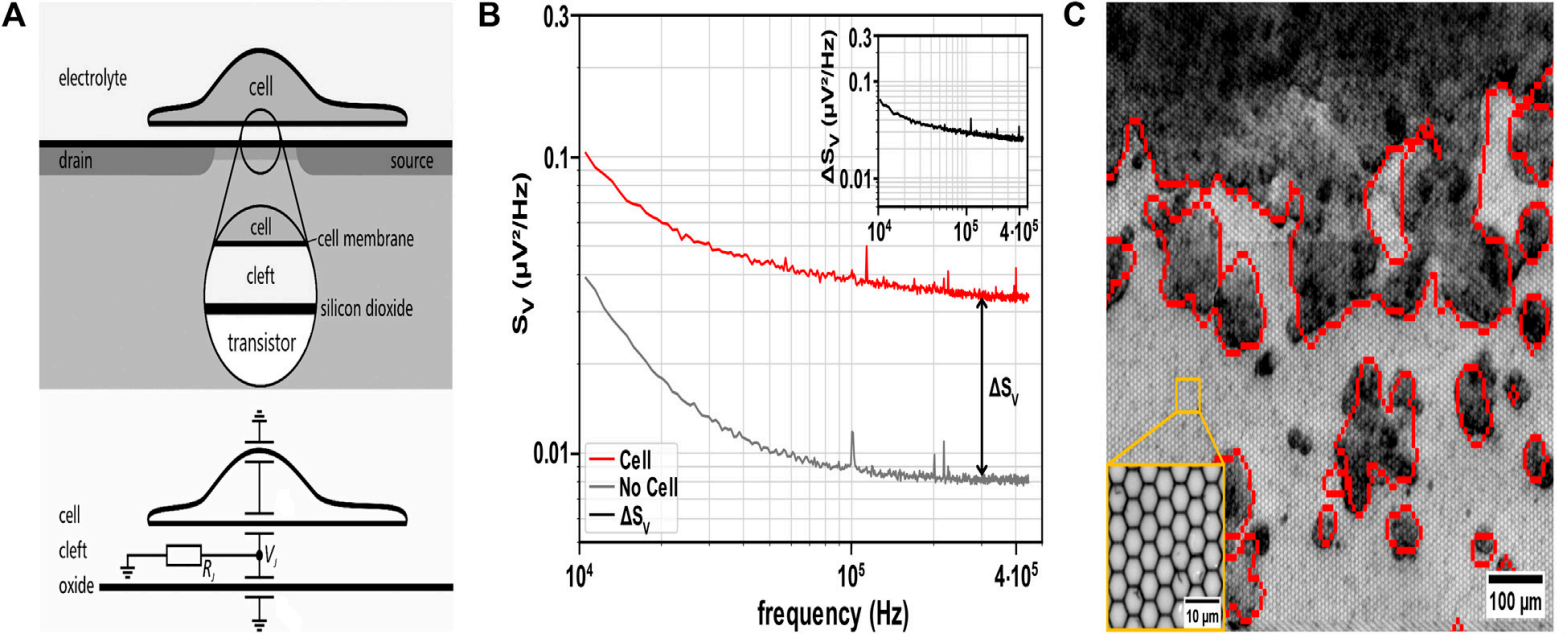
Principle of non-invasive detection of cell attachment using cell adhesion noise spectroscopy. **(A)** Top: Schematic cross-section of a cell adhered to the coated oxide surface of the recording sensor. The sensor comprises an open-gate field-effect transistor and is in contact with the electrolyte beneath the cell to record the extracellular voltage in the cleft between the cell and the chip. Bottom: Equivalent electric circuit of the cell-chip interface. The ionic current through the adherent cell membrane flows along the electrical cleft resistance R_J_ and enhances the voltage noise V_J_ (Fromherz, 1999), which characterizes the thermal noise of cell adhesion. Schematic and equivalent electric circuit adapted from (Voelker and Fromherz, 2006) with permission from SciPris on behalf of the American Physical Society. **(B)** Cell identification via cell adhesion noise (CAN) spectroscopy. A sensor must exceed the sum of ΔS_V_ and the values of S_V_ of bare sensors (i.e., background noise) to detect an adherent cell (S_V_ analyzed at 300 kHz). Inset: Cell adhesion noise ΔS_V_ obtained by subtracting S_V_ (bare sensor) from total S_V_ (red trace). **(C)** Overlay of electrically identified HT-29 cells (red contours) with brightfield microscopic image of the CMOS MEA with adherent HT-29 cells (dark grey/black: cell clusters, light grey: sensor sites in the background). Inset: ×50 magnification of sensor sites on the MEA.

The CMOS MEA biosensing platform CAN-Q Chip comprised 256 × 384 capacitive recording sites with a pitch of 5.6 µm × 6.5 µm covering an active area of 1.6 mm × 2.5 mm as described in (Vallicelli, De Matteis, et al., 2018; Vallicelli, Fary, et al., 2018). The sensor array is covered with a 30 nm ALD-TiO_2_ top oxide layer. A Perspex culture chamber is glued on the chip, exposing the recording site arrays to cell culture and medium (Zeitler and Fromherz, 2013). The recording transistor features an open gate between the source and drain contacts, which is insulated from the electrolyte by an oxide layer to prevent electric or ionic charge transfer (Figure 1A, top) (Zeitler et al., 2011; Zeitler and Fromherz, 2013; Vallicelli, Fary, et al., 2018). A local voltage change in the electrolyte-filled cleft between the cell and the recording site modulates the source-drain current. Prior studies on neurons (Voelker and Fromherz, 2006; Zeitler et al., 2011; Zeitler and Fromherz, 2013) demonstrated how the cleft resistance R_J_ enhances the voltage noise V_J_ (Figure 1A, bottom).

We calibrated the CMOS MEA with the CAN-Q Acquisition software (Venneos GmbH) with default settings using an Ag/AgCl electrode (VWR International GmbH) as an external reference electrode in PBS (1X). The CAN-Q Acquisition software assessed the spectral power density S_V_ at frequencies between 1 kHz and 450 kHz. The CMOS MEA recordings with PBS were calibrated to characterize the sensors’ background noise.

For the cell proliferation studies, we recorded the cells after the initial overnight adhesion time to the ECM protein (as described in 2.2) at 0 h, 24 h, 48 h, and 72 h. For the drug treatment studies, we changed the cell culture medium with a 5-FU-supplemented medium and recorded at 0 h, 24 h, 48 h, and 72 h (described in 2.2 and 2.3).

### 2.5 CASY cell counter and analyzer

The viability of the cells was additionally quantified using the CASY Cell Counter and Analyzer (OLS OMNI Life Science GmbH & Co. KG). CASY counts and analyzes the cells’ viability using Electronic Current Exclusion (Lindl et al., 2005) and Pulse Field Analysis. The cell size and viability are detected based on the electrical resistance of the cell membrane (Weinreich et al., 2014; Grintzalis et al., 2017).

We collected two samples for each chip: 1) cells attached to the chip and 2) cells floating in the culture medium (i.e., supernatant). The first cellular fraction was detached from the chip using 100 µL Accutase (Sigma-Aldrich GmbH) as a mild-acting enzyme (Quan et al., 2013; Chen et al., 2015; Skog et al., 2019; Nowak-Terpiłowska et al., 2021; Lai et al., 2022) after 10 min incubation at room temperature. The second cellular fraction was isolated from the medium. For that, we collected the chips medium and centrifuged the samples at 800 rpm for 5 min. Then, we removed the supernatant and resuspended the pellet in a 100 μL cell culture medium. To do the CASY measurements, 100 μL of each sample was diluted with 10 mL of CASYton buffer and loaded for measurement. A sample consisting only of medium without cells served as blank. The data were collected with a custom CASY^®^ Excel program (Innovatis AG) and analyzed with GraphPad Prism 10 as described above.

### 2.6 Data processing for CMOS MEA noise analysis

The S_V_ was analyzed as an electrical image with image processing techniques using the OpenCV Library via custom Python scripts. Firstly, we subtracted the S_V_ of the bare electrodes (i.e., background noise) from the proliferation and drug treatment recordings to extract the cell adhesion noise (CAN), assuming uncorrelated noise sources (Zeitler et al., 2011). Secondly, the electrical image was grey-scaled and blurred with a Gaussian Blur filter to reduce the amount of image noise (i.e., single-pixel errors) to enhance the identification of objects of interest (i.e., cells and cell clusters) (Haralick and Shapiro, 1992). Thirdly, Otsu’s Binarization method determined the threshold for cell segmentation (Otsu, 1979). Finally, morphological operations (i.e., opening, closing) were performed to fine-tune the object detection and recognition. For drug treatment studies, we assumed that unhealthy or dead cells detach from the MEA (Frisch and Francis, 1994; Frisch and Screaton, 2001). This leads to a decline in adhesion noise that does not differ from the background noise. After these imaging processing steps, the number of sensors detecting cells estimated the cell-covered area.

### 2.7 Microscopy validation

Brightfield images with an upright light microscope (Zeiss Axioplan, ×10 objective, Carl Zeiss AG) related the electrically estimated cell positions to ground truth. For the CMOS MEA imaging, we reconstructed the whole microscopic image from individual image sections with the Fiji (Fiji is just ImageJ, version 1.54f, RRID: SCR_002285) plugin “Stitching” described in (Preibisch et al., 2009). Afterwards, we segmented the cell-covered area with the plugin “Trainable Weka Segmentation.” Brightfield images of 96-well plates were taken with an inverted microscope (Zeiss Axiovert 35, Carl Zeiss AG) with a ×10 objective.

## 3 Results

In this study, we assessed the effects of chemotherapeutic treatment with 5-Fluorouracil (5-FU) on 2D cell cultures using the label-free cell adhesion (CAN) analysis. The proliferation status and 5-FU-induced changes of colorectal cancer (CRC) cell line HT-29 and human dermal fibroblasts (HDFs) interfaced with the capacitive recording sites of the CMOS-based microelectrode arrays (MEAs) were recorded and analyzed over several days and across multiple MEAs.

### 3.1 Label-free detection of adherent cells by adhesion noise spectroscopy

To identify adherent cells, we recorded the voltage generated in the cleft between the cell membrane and the planar oxide surface. Previous studies demonstrated that the noise of the cleft can be used to infer the presence of adherent cells (Voelker and Fromherz, 2006; Zeitler et al., 2011; Zeitler and Fromherz, 2013). Details of the detection technique are described in the method section. We assessed voltage fluctuations in the adhesion area of non-electrogenic cells (like the CRC cell line HT-29 and HDFs) cultured on the MEA recording sites (i.e., electrolyte-oxide-silicon field-effect transistors) (Figure 1A). The resistive cleft below non-electrogenic cells gives rise to the adhesion voltage noise, allowing for distinguishing this value from that of a bare sensor site (Rocha et al., 2016; Ell, Zeitler, et al., 2023) (Figure 1B). Adherent cells are detected by estimating the S_V_ spectrum. Comparing spectra from uncovered with cell-covered sensor sites, S_V_ exhibits elevated values with cells attached, which is attributed to the resistive cleft. To assess the values of the resistive cleft from the voltage noise spectral power density (S_V_), we compared the estimate of electrical imaging to brightfield microscopy by overlay (Figure 1C). Figure 1B illustrates that cells adhered to sensor sites give rise to an increased S_V_ across the entire frequency spectrum. Subtracting the S_V_ of a bare sensor from the estimated S_V_ in culture (i.e., sensors exposed to the electrolyte) provides the adhesion noise ΔS_V_ (Figure 1B inset), assuming that the two noise sources are independent (Zeitler et al., 2011). Here, we identified the frequency range with a flat adhesion noise spectrum indicating a frequency-independent (i.e., resistive) cleft from 100 kHz to 450 kHz (Voelker and Fromherz, 2006). We selected a frequency value in the middle of this frequency range, i.e., 300 kHz, for all future analyses of ΔS_V_. From the adhesion noise plateau with an average amplitude of ΔS_V_ = 0.027 µV^2^/Hz, we estimated the cleft resistance using Eq. 1 with a thermal energy k_B_T at incubation temperature (Zeitler and Fromherz, 2013).

$$R_{cleft}=∆S_{V}/\left(4k_{B}T\right)$$ (1)

Equation 1: Cleft resistance of the cell-transistor interface derived from noise spectrum. ΔS_V_ denotes the cell adhesion noise, k_B_T refers to the thermal energy with the Boltzmann constant k_B_ and incubation temperature T (Zeitler et al., 2011).

The cleft resistance R_cleft_ = 1.58 MΩ aligns with previous studies on neurons (Voelker and Fromherz, 2006). 1/f noise dominated the CAN for the S_V_ data below 10 kHz frequency, which is attributed to the intrinsic setup noise and not used for further analysis here (Haartman and Östling, 2007; Viswam et al., 2018; Lausen et al., 2022). Undetected small structures on the sensor surface indicate poor cell attachment or dead cells. The exemplary Figure 1C and parts of Figure 3 demonstrate that adhesion voltage noise is a valuable parameter for the label-free detection of adherent cells. In the following, we assess to what degree ΔS_V_ changes upon application of 5-FU to the cell culture.

### 3.2 Constant cell adhesion noise across culture time and cell types indicates stable adhesion

**FIGURE 2:**
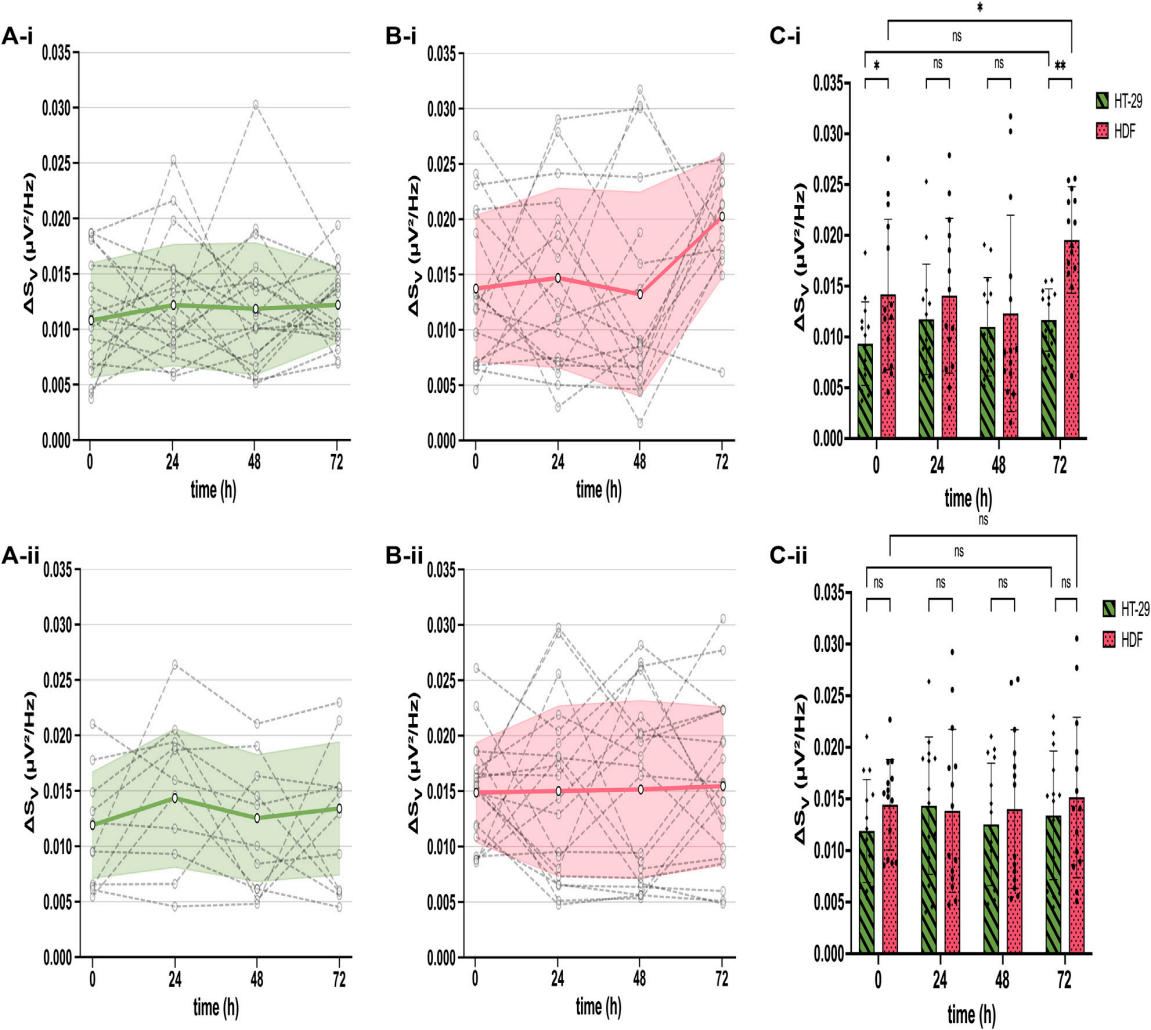
Stable cell adhesion inferred from mean ΔS_V_ across different cultures (HT-29 and HDF), time points in culture, and different chips. **(A)** Constant ΔS_V_ over time for untreated **(A-i)** and 5-FU-treated HT-29 cells **(A-ii)**. Each dashed symbol represents the mean ΔS_V_ calculated for all cell-detecting sensors on one chip. The dashed lines between symbols connect recordings obtained with the same chip. The bold green line constitutes the mean of all chips and the standard deviation of ΔS_V_ is shown as error bars. **(B)** ΔS_V_ for each chip (dashed lines with symbols) and mean with standard deviation error bars (bold pink line) of all chips over time for both untreated **(B-i)** and 5-FU-treated HDFs **(B-ii)**. **(C)** Bars: mean ΔS_V_ for untreated (green) and 5-FU treated (pink) HT-29 cells **(C-i)** and for HDFs **(C-ii)**, respectively. Dots: ΔS_V_ for each chip. Two-way ANOVA for statistical difference in ΔS_V_ between HT-29 cells and HDFs. Asterisks: ns, not significant. *, *p* ≤ 0.05. **, *p* ≤ 0.01.

After extracting the cell adhesion noise (CAN) ΔS_V_ from the total voltage noise, we compared ΔS_V_ across sensors, time, cell types, and different CMOS MEAs employed at untreated and drug-treated (5-FU) states. Therefore, we (i) identified adherent cells by their ΔS_V_, (ii) calculated the mean ΔS_V_ averaged over thousands of cell-identifying sensors (Figures 2A, B, grey dashed dots), (iii) plotted the ΔS_V_-traces of each CMOS MEA for the cultivation time of 72 h (Figures 2A, B, grey dashed lines), and (iv) estimated the mean and the standard deviation of all chips employed for both cell types (i.e., HT-29 CRC cells and HDFs) (Figures 2A, B, mean as bold green and pink line, standard deviation as error bars). Figure 2 compares the mean ΔS_V_ (at 300 kHz frequency) of adherent untreated cell networks (top) to the mean spectrum of 5-FU-treated ones (bottom).

Untreated and 5-FU-exposed HT-29 cells exhibited a mean ΔS_V_ between 0.009 µV^2^/Hz and 0.014 µV^2^/Hz with slightly higher variations of treated cells (Figure 2A). The ΔS_V_ mean for untreated and 5-FU-treated HDFs remained constant until 48 h of cultivation between 0.012 µV^2^/Hz and 0.014 µV^2^/Hz (Figure 2B). After 72 h cultivation, ΔS_V_ for untreated HDFs went up to 0.020 µV^2^/Hz with a statistical difference of *p* ≤ 0.01 (Figure 2C) while remaining constant for cells in 5-FU (Figures 2B, C). This longitudinal comparison demonstrates a stable interfacing for 72 h of the two cell cultures under test. We could not detect any significant differences except for HT-29 cells at 0 h and 72 h, which will be addressed in the discussion section. Given that ΔS_V_ remains stable for adherent cells, we simplify our analysis and assign sensors with adherent cells as “positive” sensors and with no cells as “negative.” The total fraction of “positive” sensors is used in the following to quantify cell proliferation on a coated CMOS MEA surface.

### 3.3 Assessment of cell proliferation on coated substrates

Since the values of ΔS_V_ do not change over time upon application of 5-FU (Figure 2), we assessed an additional feature provided by the cell-detecting sensors on the CMOS MEA: the cell-covered area. If the sensor area covered with adherent cells changes in control conditions in a different way than the cell-covered area upon treatment, we would have identified a potential indicator of the proliferation status. In the first experiment, we sought coating conditions where the cell-covered area increases over time.

**FIGURE 3:**
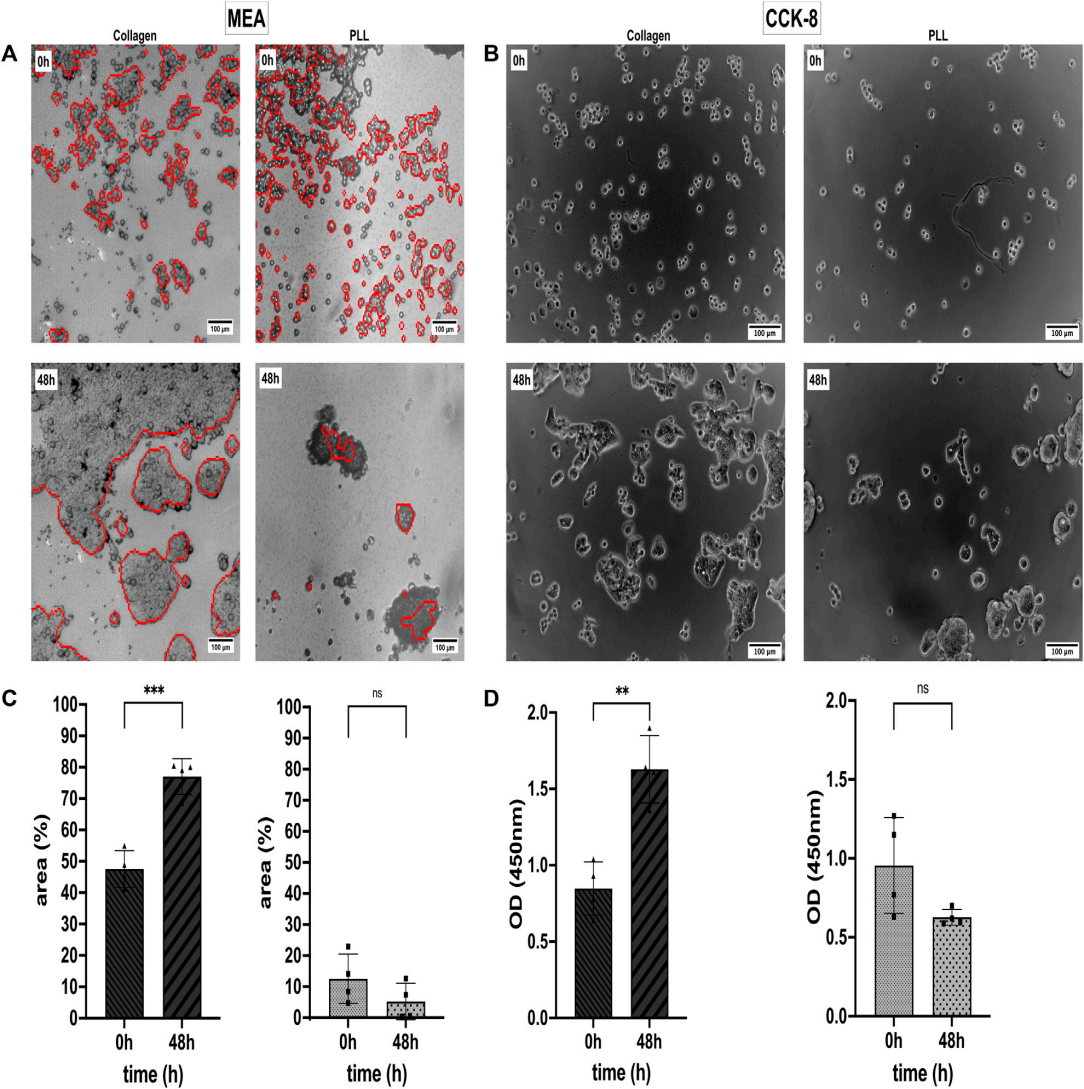
Cell attachment, viability, and proliferation status of the colorectal cancer (CRC) cell line HT-29 on Collagen type I and Poly-L-lysine (PLL). **(A)** Cell adhesion noise (CAN) spectroscopy electrically images adherent cells (red contours) on CMOS-based microelectrode arrays (MEAs). Electrical and brightfield microscopy (greyscale) images overlay for optical validation of the electrical data. **(B)** Optical study of HT-29 proliferation in 96-well plates using Collagen and PLL coating. **(C)** Mean area covered by adherent cells on Collagen (left) and PLL (right). Statistical comparison: unpaired *t*-test. **(D)** Analysis of HT-29 Cell Viability via CCK-8 assay. As in **(B)**, the left subplot shows cell viability on Collagen-I-coated substrate and the right subplot shows cell viability on a PLL-coated substrate. Statistics: unpaired *t*-test. Asterisks denote statistical significance of the indicated comparisons (*n* = 4; ns, not significant. *, *p* ≤ 0.05. **, *p* ≤ 0.01. ***, *p* ≤ 0.001. ****, *p* ≤ 0.0001).

We analyzed the Collagen and PLL effects on the viability and proliferation capacity of the CRC cell line HT-29 (Prockop and Kivirikko, 1995; Vitale et al., 2018). For that, the HT-29 cells were cultivated in a 2D monolayer in Collagen and PLL-coated 96-well plates and on CMOS MEA. Data from CCK-8 proliferation assay and CAN spectroscopy CMOS MEAs were collected. Cell Viability in %, optical density (OD), and the cell detection positive area per chip were quantitatively examined, as shown in Figure 3. Electrical imaging (red contours) was overlayed with brightfield microscopy imaging (grey background) (Figure 3A) and Figure 3B shows brightfield microscopy images of the cells in 96-well plates. After 2 days of cultivation, the HT-29 cells detached from the PLL-coated sensors while forming clusters and proliferating on Collagen-coated sensors, the same for cells in 96-well plates. We could draw two conclusions from the estimated cell detection positive area (Figure 3C): (i) initial HT-29 cell attachment is better in Collagen-coated plates (cell-covered area at 0 h: 47.5%) than in PLL-coated ones (cell-covered area at 0 h: 12.5%) and (ii) cell attachment dropped for cells growing on PLL after 2 days of cultivation (cell-covered area decreased at 48 h to 5.2%) whereas Collagen enhanced cell attachment and proliferation (cell-covered area at 48 h: 77.0%).

The CCK-8 assay allows us to evaluate the % of metabolic active cells in culture. Dehydrogenases in alive cells can reduce WST-8 [2- (2- methoxy-4-nitrophenyl)-3-(4-nitrophenyl)-5-(2, 4-disulfophenyl)-2H-tetrazolium, monosodium salt] to a water-soluble orange dye known as formazan. The amount of formazan produced correlates with the number of viable cells and can be measured with an absorbance microplate reader at 450 nm (Cai et al., 2019). In Figure 3D, it is shown that the OD increased by 1.9-fold from 0.85 at 0 h to 1.63 OD after 48 h of cultivation on Collagen. For cells growing on PLL, the OD dropped not significantly by 1.9-fold from 0.96 at 0 h to 0.63 OD after 48 h (Figures 3B, D).

HT-29 cells growing on Collagen showed constant ΔS_V_ values over time, but the cells’ ΔS_V_ showed variations when growing on PLL-coated MEAs (Supplementary Figure S1). Supplementary Figure S2 relates the two coatings (i.e., Collagen and PLL) to uncoated 96-well plates as control group with statistical significance *p* ≤ 0.01 at initial cell attachment (0 h) and *p* ≤ 0.0001 after 48 h of cultivation. Supplementary Figure S3 compares cell segmentation of electrical imaging with brightfield microscopy imaging with non-significant differences in the cell-covered area of the CMOS MEAs but slightly higher rates for microscopic images.

### 3.4 Assessing the effect of the chemotherapeutic drug 5-FU on cell adhesion

Based on the positive adhesion results, we used Collagen-I to evaluate the response of colorectal cancer cells HT-29 and non-cancerous HDF cells to the anti-cancer drug 5-FU. 5-FU has a cytotoxic effect on HT-29 cells, leading to cell apoptosis (Mhaidat et al., 2014). Consequently, cells undergoing cell death metabolize less or no WST-8 dye, which results in lower optical density (OD) for CCK-8 assay. Similarly, these cells detach from the MEA and will not be detected by the sensors. Hence, we correlated cell viability and apoptosis with OD and the area that is covered by adherent cells (Ell, Bui, et al., 2023). CASY recorded the cell viability of cells attached to the CMOS MEA surface and cells floating in the cell culture medium.

**FIGURE 4:**
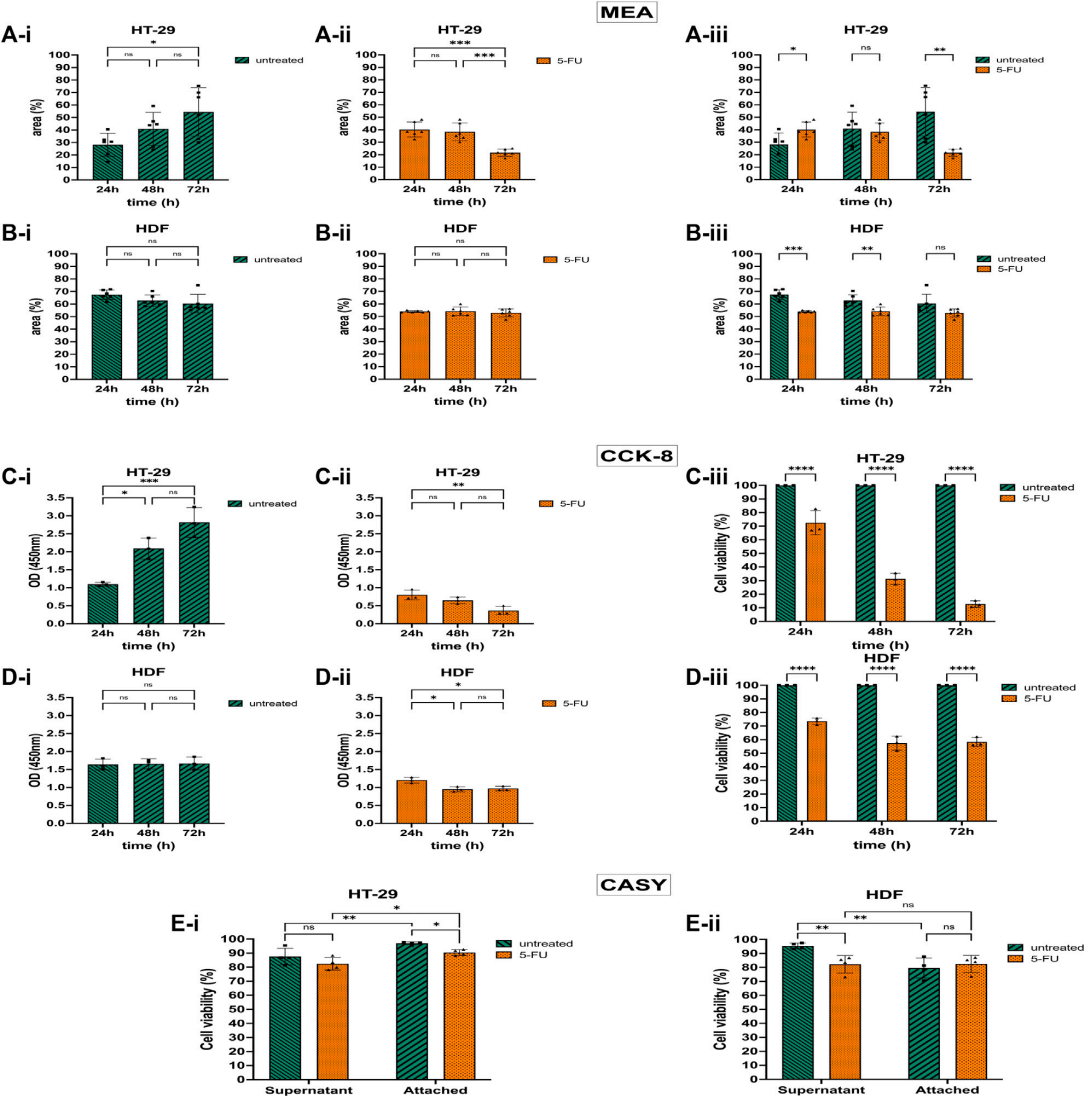
5-Fluorouracil (5-FU) with cytotoxic and detachment-enhancing effect on the colorectal cancer cell (CRC) line HT-29 but without effect on human dermal fibroblast (HDF) cell viability and proliferation. **(A)** 5-FU effects via CAN spectroscopy on HT-29 cells cultivated on the CMOS MEA (analyzed at 300 kHz). Untreated HT-29 grew on the CMOS MEA and the cell-covered area rose **(A-i)**. After 5-FU treatment, the detection positive area declined after 72 h of cultivation **(A-ii)**. Statistics **(A-i,A-ii)**: one-way ANOVA. Statistical difference of the cell-covered area between untreated and treated HT-29 **(A-iii)**. Statistics **(A-iii)**: two-way ANOVA. **(B)** 5-FU effects via CAN spectroscopy on HDF cells cultivated on the CMOS MEA (analyzed at 300 kHz). Untreated and treated HDFs showed a stagnant CMOS MEA area where cells are adhered to **(B-i,B-ii)**. Statistics **(B-i,B-ii)**: one-way ANOVA. CMOS MEA area covered by HDFs **(B-iii)**. Statistics **(B-iii)**: two-way ANOVA. **(C)** 5-FU effects on HT-29 cells via CCK-8 assay. Untreated HT-29 cells’ optical density (OD) increased during 72 h cultivation time **(C-i)**, while 5-FU treated cells’ OD declined **(C-ii)**. **(D)** 5-FU effects on HDF cells via CCK-8 assay. For HDFs, the OD stays constant without **(D-i)** and with 5-FU treatment **(D-ii)**. Statistics: one-way ANOVA. **(C-iii,D-iii)** compared cell viability of treated cells with untreated cells as control group. Treated cells showed less cell viability for both cell types [HT-29 **(C-iii)** and HDF **(D-iii)**]. Statistics: two-way ANOVA. **(E)** Cell viability from CASY recordings of treated and untreated HT-29 in the supernatant and on the chip **(E-i)**. Same for HDFs **(E-ii)**. Two-way ANOVA. Statistical significance is indicated by asterisks (n_HT-29_ = 4, n_HDF_ = 4, ns, not significant. *, *p* ≤ 0.05. **, *p* ≤ 0.01. ***, *p* ≤ 0.001. ****, *p* ≤ 0.0001).

Untreated HT-29 cells growing on the Collagen-coated MEA proliferated and the area covered by adherent cells doubled after 72 h in culture (Figure 4A-i). However, after treating the HT-29 cells with 5-FU, approximately half of the cells detached from the chip and the cell-covered CMOS MEA area declined by 1.8-fold after 72 h exposure time (Figure 4A-ii). Figure 4A-iii shows statistical significance *p* ≤ 0.01 for untreated and 5-FU-treated HT-29 cells after 72 h with less cell-covered area (54%–22%), while a shorter exposure time to 5-FU for 24 h led to a larger cell-covered area (28%–40%). The ODs for the HT-29 cells matched the CAN-based areas: untreated HT-29 cells showed a 2.5-fold higher OD at 72 h compared with 24 h (Figure 4C-i), whereas HT-29 cells exposed to 5-FU showed a decline in OD by 2.2-fold (Figure 4C-ii). The agreement between CAN and OD value is, however, detected only after 72 h for HT-29 cells.

While 5-FU caused cell detachment on the CMOS MEA and a lowered metabolic activity in the CKK-8 assay for the HT-29 cells, the HDFs’ proliferation status was altered differently: the CMOS MEA’s positive cell detection area for untreated HDFs was on a constant level (Figure 4B-i) and also the OD did not change (Figure 4D-i). For the 5-FU-treated HDFs, the cell-covered area remained constant over 72 h (Figure 4B-ii). The OD decreased within the first 24 h of 5-FU exposure and stagnated for the remaining culture time (Figure 4D-ii). Untreated HDFs showed a slight decrease in cell-covered area (Figure 4B-i), while treated ones remained on a constant coverage over time (Figure 4B-ii). Figure 4B-iii indicates a statistical difference of *p* ≤ 0.001 for 24 h and *p* ≤ 0.01 for 48 h, but no significance for 72 h by analyzing the cell-covered area. The cell viability lowered upon 5-FU application to 74% after 24 h, 57% after 48 h, and 58% after 72 h drug exposure time with *p* ≤ 0.0001 (Figure 4D-iii).

CASY results in Figure 4E-i show elevated cell viability for untreated HT-29 cells compared with 5-FU-treated cells in the supernatant. HT-29 cells adhered to the MEA exhibited significantly higher cell viability than in the supernatant with *p* ≤ 0.01 (untreated) and *p* ≤ 0.05 (treated). CASY recorded higher viability for untreated HDFs in the supernatant than for 5-FU-exposed ones with *p* ≤ 0.01 (Figure 4E-ii). Untreated as well as treated CMOS MEA-adherent HDFs remained at the same viability level.

## 4 Discussion

In this study, a label-free, time-continuous electric detection method is presented, which enabled the assessment of chemotherapeutic effects on two different cell cultures (colorectal cancer (CRC) cell line HT-29 and human dermal fibroblasts HDFs). The electric detection of cell adhesion voltage noise has been validated by microscopy and with commercial reference methods, such as Cell Counting Kit-8 (CCK-8) and CASY Cell Counter and Analyzer.

In the following lines, we discuss the results obtained using cell adhesion noise (CAN) in comparison with the reference methods from a technical perspective and biological context. We furthermore address shortcomings and point towards improvements that may be addressed in future work.

To analyze the adhesion noise across sensors, MEA chips, time, and cell types, the CAN spectra were averaged over thousands of sensors and the standard deviation was determined for both HT-29 CRC cells and HDFs detecting sensors. Our reported cleft resistance R_J_, estimated with Eq. 1, is in accordance with prior studies on the neuron-transistor interface (Voelker and Fromherz, 2006). HDFs showed a slightly elevated CAN compared to HT-29 in both conditions (5-FU-treated and untreated). According to the model of a circular core-coat conductor of a cell-solid junction, as proposed in (Zeitler and Fromherz, 2013), ΔS_V_ is dominated by three free parameters: the sheet resistance r_J_ at low frequency (<1 kHz), the effective cell capacitance c_M_ (frequency range: 1 kHz–1 MHz) or the cytoplasmic resistivity ρ_cyt_ at high frequency (>1 MHz). Additionally, ΔS_V_ depends on the given parameters, such as the sensor radius, adhesion area, and the thermal energy k_B_T, as shown in (Zeitler and Fromherz, 2013). Given the bath resistivity of ρ_E_ = 66.7 Ω cm, we calculate the cytoplasmic resistivity to 937 Ω cm for HDFs and 803 Ω cm for HT-29. However, HDFs are bigger than HT-29 cells (Coumans et al., 2013; Tai et al., 2022) and should therefore show higher ΔS_V_ values. Further research could focus on tumor cells altering the cell adhesion molecules (Harjunpää et al., 2019) to better understand the effects on cytoplasm resistivity. Additionally, investigating the actual membrane-oxide surface distance with, e.g., fluorescence interference contrast (FLIC) microscopy could provide more insight into the sheet resistance influence (Iwanaga et al., 2001; Fromherz, 2002; Schoen and Fromherz, 2007; Zeitler and Fromherz, 2013).

The average values of ΔS_V_ did neither change over time nor upon application of 5-FU except for HT-29 cells at 0 h and 72 h. Integrins as transmembrane proteins mediate cell adhesion to the extracellular matrix (ECM) by spanning the plasma membrane (Santini et al., 2000). This cell adhesion through integrins stimulates numerous signaling pathways, including ion channel activation. Therefore, ion channels that may stochastically open and close can generate current fluctuations (Siebenga et al., 1973) giving rise to the voltage noise in the cleft. Since integrin functions can be dynamically regulated by combinations of conformational changes and cells clustering (Dedhar, 1999), initial cell attachment at 0 h can alter the CAN significantly (Figure 2C). Not only cell attachment but also cell death by cell detachment [i.e., anoikis (Frisch and Francis, 1994; Frisch and Screaton, 2001)] is regulated mainly through integrins. Hence, apoptosis-induced cell detachment is accompanied by the loss of structural links of the ECM to the cell. These active membrane processes can cause mechanical fluctuations, which are recorded with CAN spectroscopy (Seifert, 1994; Prost and Bruinsma, 1996; Voelker and Fromherz, 2006). We quantified the cells’ proliferation status on a coated CMOS MEA substrate with the total fraction of cell-detecting (i.e., “positive”) sensors. The estimated positions of the cells (“positive” sensors) on the MEA aligned with microscopic brightfield images with high accuracy (Figure 1C).

To enable favorable conditions for the cell culture on the CMOS MEAs, we first compensated for the hydrophobic surface of the MEA’s surface oxide (Bae et al., 2019) by coating it with an extracellular matrix (ECM) protein or with a synthetic polymer. Cell interactions with the extracellular matrix (ECM) are vital for cell adhesion, migration, and growth (Cruz Walma and Yamada, 2020). ECM proteins like Collagen Type I and synthetic polymers like Poly-L-lysine (PLL) ensure surface cell adhesion *in vitro* (Vitale et al., 2018). Besides, Collagen-I is the most abundant type of Collagen in the tissues and plays a main role in cell normal cell biology and metastasis (De Martino and Bravo-Cordero, 2023). The common coating polymer in neuroscientific applications, Poly-L-lysine, caused a decline in the cells’ metabolic activity, resulting in a lower optical density (OD) than cells cultivated on Collagen (Figure 3D). Previous studies indicated PLL’s cytotoxic, apoptotic, and genotoxic effects on mammalian cells (Alinejad-Mofrad et al., 2019). Additionally, ECM proteins can rescue cells from anoikis, but surfaces coated with PLL cannot (Meredith et al., 1993; Frisch and Ruoslahti, 1997). In contrast, Collagen as the major component for ECM (Kallis and Friedman, 2018) promoted cell adhesion and proliferation (Mainardi et al., 1980; Greco et al., 1998; Somaiah et al., 2015) with OD increasing over time. For the CMOS MEA recordings, we assume that unhealthy or dead cells detach from the MEA (Frisch and Francis, 1994; Frisch and Screaton, 2001) with a decline in cell adhesion noise below the cell identification threshold. Thus, proliferative cells on Collagen covered a larger area on the MEA than cells on PLL with lowered metabolic activity (Figure 3C). Brightfield microscopy confirmed the positive impact of Collagen (i.e., higher cell-covered area) and the negative impact of PLL (i.e., lower cell-covered area) on cell viability cultivated on the MEA (Figures 3A, B). An immediate future study could focus on how different coatings and coating concentrations alter cell adhesion (like PLL’s concentration-dependent cytotoxicity, as reported in (Alinejad-Mofrad et al., 2019)). Additionally, since cancer cells have an altered glycocalyx structure, cell adhesion receptors (e.g., integrins) as part of the glycocalyx could be investigated for a better understanding of ECM protein-cancer cell adhesion interactions (Burridge et al., 1988; Hollingsworth and Swanson, 2004; Xu et al., 2016; Kanyo et al., 2020). Moreover, analyzing the distribution of the chip coatings over the MEA surface (e.g., using mass spectrometric bio- and polymer analysis) could explain the variability of the initial cell attachment (Figure 3C).

Next, the area of the sensor array covered by adherent cells was related to cell proliferation as an indicator of the health status with and without drug treatment. While untreated HT-29 cells proliferated thereby increasing MEA coverage and leading to higher OD, 5-FU-exposed HT-29 cells detached after 48 h of cultivation with a significant reduction of metabolic activity via CCK-8 (Figure 4A). As opposed to the HT-29 cells, 5-FU did not cause a lowered cell proliferation on HDFs via CAN spectroscopy but remained constant (Figure 3B). The shorter doubling time of HT-29 cells (Forgue-Lafitte et al., 1989; Frant et al., 2022) compared to HDFs (Zhu et al., 2004; Endt et al., 2010; Rorteau et al., 2022) may lead to a higher 5-FU-uptake, resulting in stronger proliferation-reducing effects of drug treatment. However, the HDFs’ metabolic activity in the CCK-8 assay in the 5-FU-supplemented medium dropped after 24 h of exposure and stayed constant for the remaining time (Figure 3B). This could indicate that the primary cell line reduces its proliferative capacity but not its viability (Krtolica et al., 2001). An immediate future work could focus on drug concentration-dependent cell viability studies to calculate the GI50, TGI, and LC50 to compare with the NCI 60 cell-line screening panel (Chabner, 2016; Developmental Therapeutics Program, 2021).

We recorded the viability using CASY Cell Counter and Analyzer for the experimental end time point because significant changes in the cell-covered area occurred after 72 h of 5-FU exposure (Figure 4B). CASY recorded high viability for both cells on the MEA and in the supernatant. The high cell viability on the MEA is in accordance with the elevated S_V_ due to cell adhesion accounting for healthy status. In contrast, the high viability of the detached cells (i.e., floating cells in the supernatant) could be explained by cells being in an early stage of apoptosis. During an apoptotic process, adherens junctions are destructed, and adhesive complexes are destabilized (Suzanne and Steller, 2009). Therefore, cells start to detach but are not recorded as dead cells. Supplementary Figure S4 shows the same behavior after 5 days (i.e., 120 h) of 5-FU-treatment.

Our MEA technique provides one of the smallest electrode separations (∼6 µm) and therefore highest spatial resolution reported in literature [reviewed in (Iyer et al., 2023), but see (Laborde et al., 2015; Widdershoven et al., 2018)] enabling the non-invasive detection of non-electrogenic single cells, but also cell network studies. CAN spectroscopy electrically images only viable cells that are attached to the CMOS MEA’s oxide surface, while brightfield microscopy images all cells present on the chip adhered or not. Moreover, the method is label-free in contrast to fluorescence microscopy, which also images viable cells with subcellular resolution (Supplementary Figure S5). A related method to CAN spectroscopy is the electrochemical impedance spectroscopy (EIS) (Viswam et al., 2017; Abbott et al., 2022). While the readout (extracellular voltage) is identical for both methods, EIS requires AC stimulation and may thus be considered somewhat more invasive, depending on the implementation. In contrast, EIS offers a richer readout, given that different readout configurations (2-point, 4-point measurements) are feasible. In future work, the high spatial resolution may be explored further when analyzing the effects of different agents on the cell-covered area.

Overall, this new application of CMOS MEAs is in good agreement with the commercially available biological assay Cell-Counting Kit-8 (Figure 3B). Moreover, we gained further insight into the cells’ health status with the electrical cell counter CASY by studying the detachment behavior of cells already in the early stages of apoptosis (Figure 3C). In conclusion, CAN spectroscopy is label-free, non-invasive, and demonstrates high accordance with the CCK-8 assay over many days of culture via high spatiotemporal resolution to be used for drug screening studies. In conclusion, CAN spectroscopy combines the advantages of brightfield microscopy, impedance spectroscopy, CMOS technology, and biological assays to complement existing drug screening techniques: label-free, non-invasive, and fast electrical imaging with subcellular resolution at high accuracy over many days of culture.

## Data Availability

The original contributions presented in the study are included in the article/Supplementary Material, further inquiries can be directed to the corresponding authors.
