# Supplementary material for: Assessment of chemotherapeutic effects on cancer cells using adhesion noise spectroscopy
Source: Front Bioeng Biotechnol. 2024 May 13;12:1385730. doi: 10.3389/fbioe.2024.1385730 (PMC11128629; doi:10.3389/fbioe.2024.1385730)
Supplement: Supplementary file 1 [file DataSheet1.pdf]

Supplementary Material

1 Assessment of cell proliferation on coated substrates

1.1 Non-constant cell adhesion noise for cells on different coatings

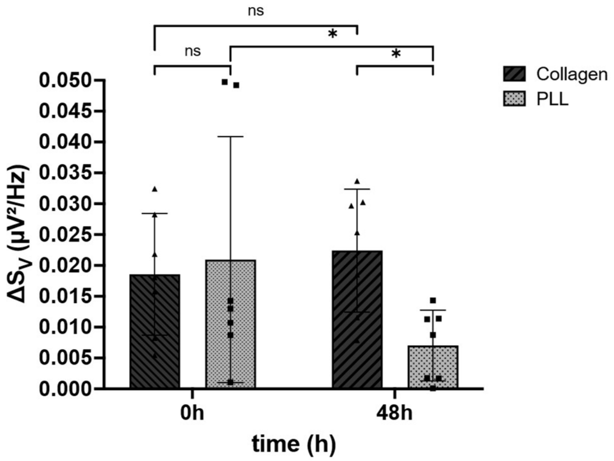

Supplementary Figure S1. Stable cell adhesion noise ΔS_V of HT-29 cells for collagen-I-coated CMOS microelectrode arrays (MEAs) and non-constant ΔS_V for cells on PLL-coated MEAs. Statistics: Two-way ANOVA, n=7. Statistical significance via asterisks: ns, not significant. *, $P \leq 0.05$.

1.2 Cell viability for different substrate coatings

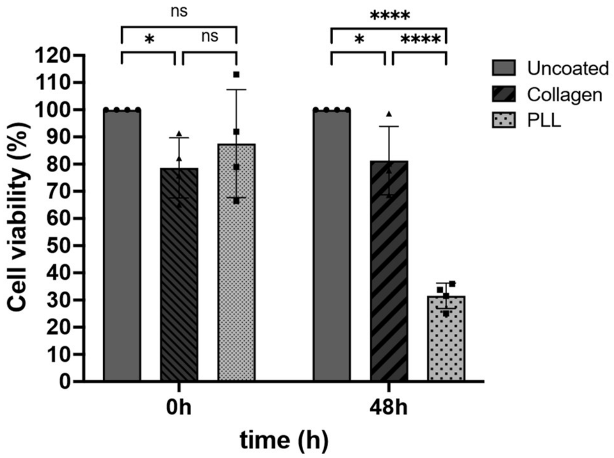

Supplementary Figure S2. Statistical analysis of CCK-8-based cell viability and OD of uncoated, collagen-, and PLL-coated 96-well plates. Cells in uncoated 96-well plates served as control group. Statistical significance via asterisks: ns, not significant. *, $P \leq 0.05$. **, $P \leq 0.01$. ***, $P \leq 0.001$. ****, $P \leq 0.0001$.

1.3 Cell segmentation of microscopy images aligns with segmentation of electrical images

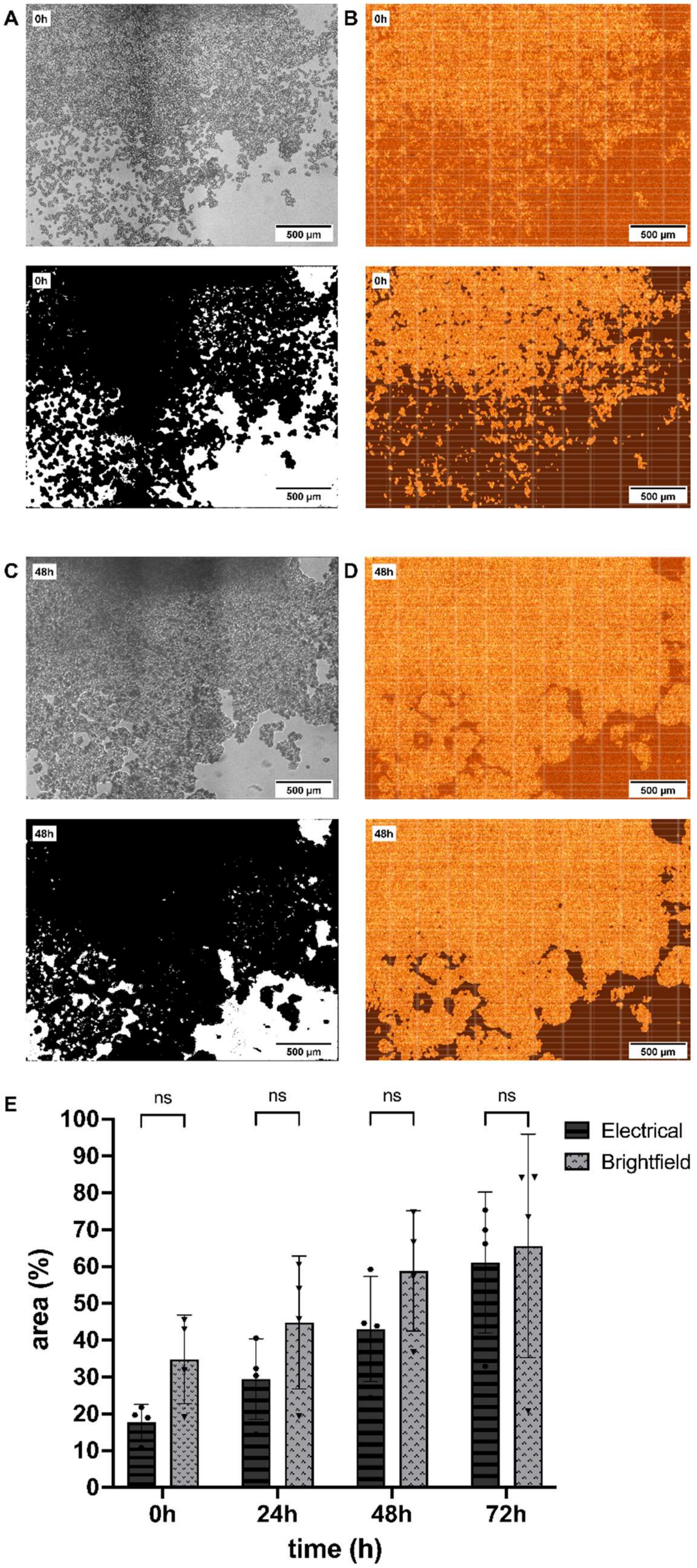

Supplementary Figure S3: Cell segmentation of brightfield (BF) microscopy images via Fiji matches the results of Python-segmented electrical images for untreated cells. **(A)** BF microscopy image (A-i) and Fiji-based cell segmentation of microscopic image (A-ii) on collagen-coated microelectrode array (MEA) at 0 h culture time. **(B)** Electrical image via cell adhesion noise (CAN) spectroscopy (B-i) and Python-based cell segmentation (B-ii); same sample as in (A). **(C)** Same as in (A) at 48 h culture time. **(D)** Same as in (B) at 48 h culture time. **(E)** Statistical comparison of the cell-covered area between Fiji-based cell segmentation of BF images and Python-based segmentation of electrical images. Statistical significance via asterisks: ns, not significant.

2 Assessing the effect of the chemotherapeutic drug 5-FU on cell adhesion

2.1 Longterm analysis of early apoptosis

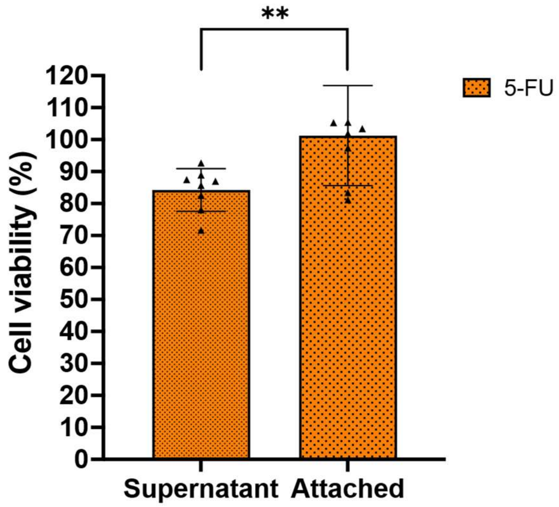

Supplementary Figure S4: Cell viability from CASY recordings of HT-29 cells in the supernatant and on the chip after 5 days (120 h) of treatment with 5-FU. Statistics: paired t-test. Statistical significance is indicated by asterisks (n_{HT-29}=4, n_{HDF}=4, ns, not significant. *, $P \leq 0.05$. **, $P \leq 0.01$).

3 Discussion

3.1 Electrical imaging of non-electrogenic cells with subcellular resolution

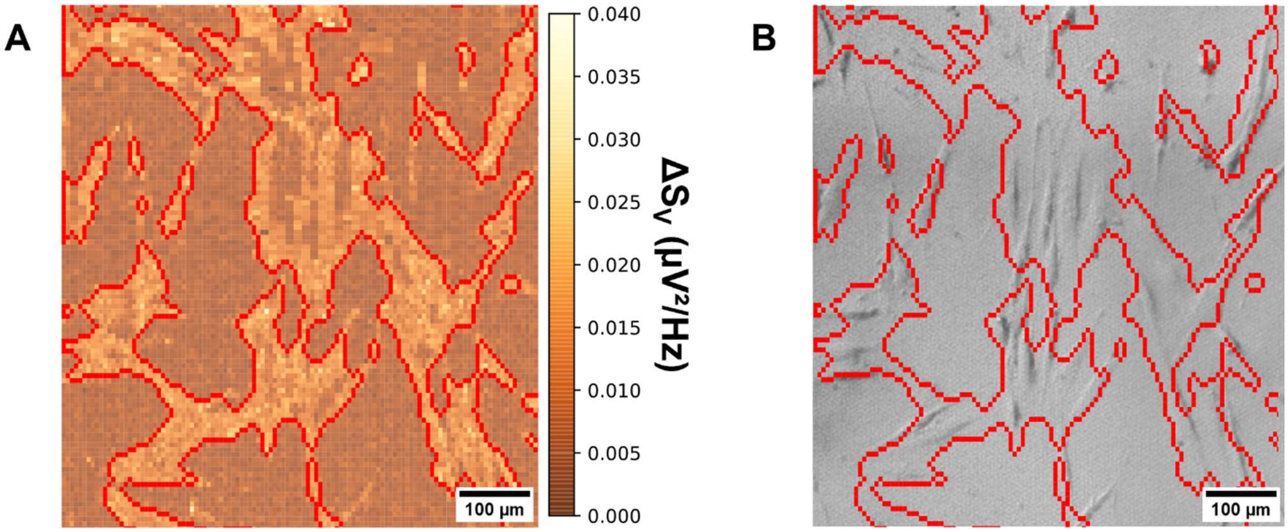

Supplementary Figure S5: Cell segmentation of electrical images via Python for untreated human dermal fibroblasts (HDFs). **(A)** Raw electrical imaging data overlayed with electrically detected cells (red contours). Background noise: brown, cell adhesion noise (ΔS_V): orange. **(B)** Brightfield microscopy image overlayed with electrically detected cells (red contours).
